# Burden of melioidosis in India and South Asia: Challenges and ways forward

**DOI:** 10.1016/j.lansea.2022.03.004

**Published:** 2022-05-05

**Authors:** Prasanta R Mohapatra, Baijayantimala Mishra

**Affiliations:** a Department of Pulmonary Medicine & Critical Care, Bhubaneswar, India; b Department of Microbiology, All India Institute of Medical Sciences, Bhubaneswar, India-751019

**Keywords:** Melioidosis, Burkholderia pseudomallei, South Asia, India, Challenges, Prevention

## Abstract

Melioidosis is caused by the environmental bacterium *Burkholderia pseudomallei*. South Asia is estimated to have 44% of the global disease burden. Among South Asian countries, Bangladesh and Sri Lanka are considered endemic for melioidosis; a few cases have been reported from Nepal, and a few imported cases from Pakistan have also been reported. India has experienced an increase in numbers of melioidosis cases in the recent years. The bacteria is inherently present in the soil and enters the human body via skin abrasions, inhalation, or ingestion. As clinicians are often ignorant about the similar characteristics of this disease and several other common tropical diseases, it causes a major delay in the timely diagnosis and management. The organism is easily mistaken as *Pseudomonas spp* in microbiology laboratories and may be dismissed as a common laboratory contaminant. The poor diagnostic sensitivity of blood culture also leads to missed diagnosis. Hence, both clinical ignorance and missed laboratory diagnosis have misrepresented melioidosis as a rare entity. The key preventive interventions are avoiding contact with loose and muddy soils of meliodosis-endemic areas, and provision of safe drinking water. The present article describes the various possible attributes for melioidosis underdiagnosis and the challenges of improving the diagnosis in conjunction with viable solutions.

**Funding:**

None.

## Introduction

Melioidosis, a disease of tropical climates, is caused by an environmental Gram-negative bacterium *Burkholderia pseudomallei*. In the presence of known risk factors like diabetes mellitus and chronic renal disease, clinical clues include presentation during the monsoon months and a history of soil exposure with or without skin abrasion. Clinical episodes of pneumonia, septicaemia, multiple subcutaneous or deep-suppurative abscesses (spleen, prostate, submandibular and cervical lymph nodes affliction) are prevalent. Melioidosis is attributed to nearly 20% of community-acquired bacteraemia.^1^ The usual culture report of 'Pseudomonas like organisms resistant to aminoglycosides, polymyxin or colistin' in a patient unresponsive to conventional treatment in endemic countries is a suspect.^1^^,^^2^ Increasing cases of melioidosis have been detected in the previous few years owing to an improvement in diagnostic facilities. The possibility of higher prevalence of melioidosis in India, underdiagnosis, under-reporting of cases have been pointed out as early as 2005; however, the entity continues to remain underdiagnosed and misdiagnosed due to a lack of knowledge and awareness and continues to be considered as a rare disease.^3^ A correct diagnosis and management can be made with appropriate measures, and the true disease burden can be ascertained to address the morbidity and mortality.

## The burden of melioidosis

A modeling study in 2016 estimated that about 165,000 people got infected with melioidosis every year worldwide, of which South Asia alone contributed to 44% of the global burden of melioidosis.^4^ This figure excludes the major segment of melioidosis that goes “severely under-reported in 45 countries where it is endemic”.^4^ The estimated global burden of melioidosis during 2019 in the form of disability-adjusted life-years (DALYs) was 4·64 million.^5^ The years of life lost(YLL) accounts for nearly 99% of the figure as a result of higher mortality with melioidosis.^5^ Melioidosis mainly affects rural populations with a poor socioeconomic background in low and middle-income countries; reflecting the reason of most of the deaths due to melioidosis occurs in low-income countries.^4^

Among the South Asian countries, Bangladesh and Sri Lanka are endemic for melioidosis; some cases have been reported from Nepal. Various publications from Sri Lanka and Bangladesh have selectively reported over 220 and 89 cases, respectively.^6^^,^^7^ Studies from Pakistan demonstrate the presence of *B. pseudomallei* in soil samples collected from Lahore.^4^ The first case from Pakistan was reported in 1970 in a person with diabetes with a discharging sternal sinus and splenomegaly, was being investigated for the previous four years in Pakistan and was incidentally diagnosed in London after entry to the United Kingdom.^8^ Among the imported cases in England and Wales prior to 1999, nearly half were from South Asia (Bangladesh, India and Pakistan), the majority from Bangladesh.^9^ The first reported case from India was a 40-year old Scottish mining engineer working for two decades in India, who had developed pneumonia and a splenic abscess; he was diagnosed in 1951 and died soon thereafter.^10^ A series of cases from India were reported in 1996 who had never travelled abroad.^3^ From these instances, it is clear that melioidosis has been prevalent in many parts of South Asia for more than five decades but remains underdiagnosed and under-reported.^11^ Recently, a series of deadly melioidosis cases in the USA were traced to aromatherapy spray of “lavender and chamomile” imported from India, underlining the potential of transmission of *B. pseudomallei* through commercial liquid products.^12^ Overall mortality rates from melioidosis were 20% in Sri Lanka^13^ and 27% in Bangladesh.^14^ This disease is also widely prevalent in Southeast Asian countries like Myanmar, Indonesia, Malaysia, Singapore, Thailand and Vietnam. In the present article, we focus on the current challenges in diagnosis and control of melioidosis in South Asia, highlighting particularly the Indian scenario.

## Major challenges

The reasons for the underdiagnosis of melioidosis is multifactorial. The reported cases in India are apparently sporadic and inconsistent due to the lack of awareness about its manifestation among clinicians and scarce laboratory support. The bacteria are endemic in tropical countries with lower socioeconomic conditions. Death due to melioidosis occurs primarily in the villages of low- and middle-income countries(LMIC), where the patients approach the health care facility in the terminal, critical phase of the disease.^15^ One of the reasons the disease remained neglected is that melioidosis is unlikely to spread from human to human,^2^ like the COVID-19 outbreaks. Still, it continues to inflict higher mortality in endemic areas. But as an infectious disease, both incidence and mortality can be markedly reduced with global control measures 0.^15^

The reporting of melioidosis is mainly dependent on the laboratory setup to confirm the diagnosis. Geographical mapping of the organism distribution is not possible because of the nonavailability of the bacterial culture of clinical specimens in the rural endemic area where melioidosis is common. Again, isolating bacteria from the environmental samples is challenging, creating difficulty in establishing the actual distribution.^16^ Most Indians live in rural areas and work in the agriculture fields, and have a high chance of acquiring the infection by direct contact with water and soil. They hardly have access to good hospitals where microbiology laboratories and physicians establish diagnoses. Low awareness about the clinical disease among physicians and microbiologists further leads to overlooking of the diagnosis and inappropriate drug management.^17^

### The rise of cases in recent years

**Figure 1:**
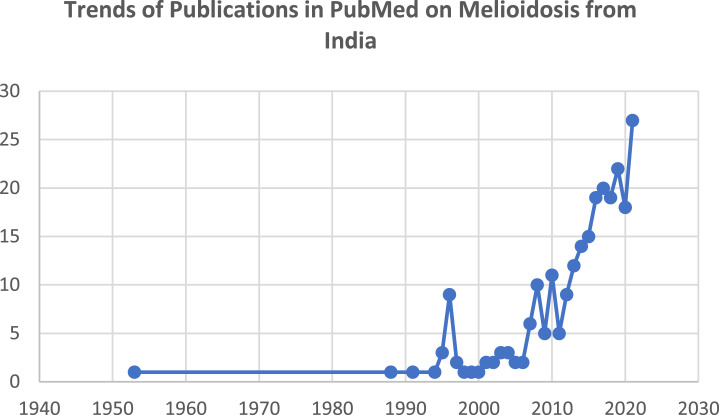
**Publications in PubMed on melioidosis from India.** Since the last decade, there has been an increasing trend of case reports, case series, and publications. Nearly half of the reports from India have been reported in the previous five years and over 70% during the last decade.

Presently, the only possible source of the data on melioidosis in the South Asian region is published case reports and case series; these are undoubtedly the tip of the iceberg.^17^ We searched PubMed with the keywords ‘melioidosis’ and ‘India’. As per available data therein, nearly half of the reports from India have been reported during the last five years and over 70% during the previous decade. Indeed, nearly 1550 out of 1700 cases (>90%) published so far from India have been reported during the last ten years. With the expansion of lab services and awareness, the annual incidence is rapidly increasing. The highest number of annual cases (nearly 600 patients from recent years) has been reported during 2021. Thus, the disease is now surfacing and being detected avidly in India. The exponential growth of reporting through publications, particularly in the previous five years in India, is depicted in the graph (Figure-1).

**Figure 2:**
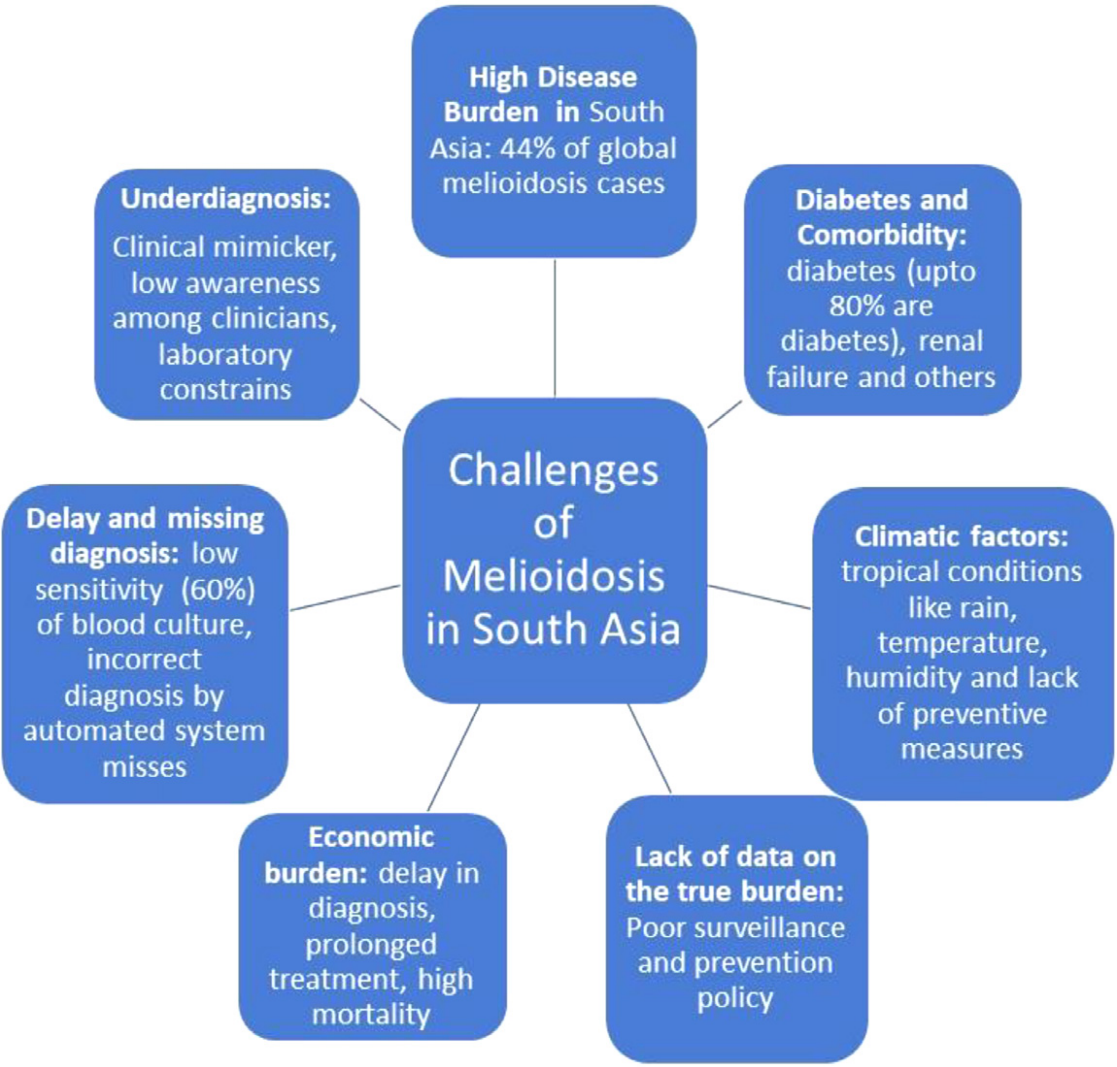
**Challenges of melioidosis control in South Asia****^2^****^,^****^5^****^,^****^13^****^,^****^14^****^,^****^20^****^,^****^23^** Lack of actual data, favourable climatic factors, a swift increase of cases, underdiagnosis, delay in diagnosis, economic burden due to morbidity, mortality and long duration of antimicrobial treatment are some of the challenges.

However, these reports are mainly from South India and sporadically reported from eastern and western parts of the country. But there are occasional reports from coastal states. So, there is a high possibility that cases are neither diagnosed nor reported from some tropical areas of the country. Moreover, published reports are just an indirect reflection of cases as all diagnosed cases are not published. Hence, the actual cases are much higher than realised. Major challeges of melioidosis control in South Asia are discuused in Figure 2.

### Great mimicker of other diseases

The signs and symptoms are non-specific and overlap with several diseases, including tuberculosis. Thus melioidosis, ‘the great mimicker’ of many diseases, is grossly underdiagnosed and underreported across the tropics, including India.^3^ In South Asia, where tuberculosis is widely prevalent, any disease like tuberculosis is misdiagnosed, leading to a delay in proper management.

### Delay and missing the diagnosis

The presence of common broad-based signs and symptoms, the diseases resemble clinically with other conditions. Clinical diagnosis is usually missed or delayed because of a lack of awareness or familiarity with this disease. Additionally, inappropriate use of broad-spectrum antibiotics suppresses bacterial isolation but does not eradicate the diseases.^2^ *Burkholderia pseudomallei* looks like Pseudomonas sp, a common contaminant and is easily misidentified in microbiology laboratories. The diagnosis is often overlooked because of blood or tissue culture's low diagnostic sensitivity (nearly 60%). Therefore, nearly 40% of diagnoses are missed initially.^2^

### The constraint of the laboratory in diagnosis

The colonies of *B. pseudomallei* often appear wrinkled and assumed to be environmental, aerobic spore-bearing contaminants and usually discarded. Gram-negative, oxidase-positive, non-lactose fermenting bacilli is traditionally considered as ‘Pseudomonas.’ Even the newer automated systems like VITEK-2, MALDI-TOF mass spectrometry and molecular technique like 16S ribosomal RNA gene sequencing may not be reliable for routine identification of *B. pseudomallei*,^12^ particularly in lack of proper training and technology.^18^ The molecular techniques are not yet routine in most places in developing countries, and several succumb due to misdiagnosis and incorrect treatment. The organism *B. pseudomallei* is intrinsically resistant to penicillin, ampicillin, first and second-generation cephalosporins and aminoglycosides like gentamicin and tobramycin, streptomycin, and polymyxin.^19^ Several patients remain on antitubercular drugs, while others are treated with high-end antibacterial agents like carbapenems with inadequate and shorter regimens leading to temporary suppression and recurrence of infection.

### High mortality and relapse

In addition to low sensitivity (60·2%) of the culture of melioidosis,^2^ case fatality is seen in up to 50% of cases^5^ and relapse rate is 20% in some settings.^20^ The need for prolonged (6 months) antimicrobial treatment entails a high cost of health care.^2^^,^^5^

### Diabetes and comorbidities

The disease mainly affects adults (median age −5th decade) with poorly controlled diabetes in the Indian subcontinent – the diabetic capital of the world.^2^^,^^5^^,^^21^ Up to 80% of melioidosis cases in India, Sri Lanka, and Bangladesh are diabetics.^13^^,^^14^^,^^21^ Patients with chronic renal disease and immunocompromised are similarly more prone to infection.^22^

### Clinical latency

*Burkholderia pseudomallei* infection may present as acute or chronic in most cases or remain latent in a dormant state. This disease may remain sub-clinical in immunocompetent individuals who cleared the infection due to immunity. In addition, wide use of broad-spectrum antibiotics at suboptimal doses suppresses the bacteria without eradicating the infection, leaving the chance of recurrence from its latency even after years.^2^^,^^24^^,^^25^

### Climatic and occupational predilection

Melioidosis, as a disease, has strong associations with tropical climatic conditions - rainy, hot and humid. Coastal areas' environmental and demographic factors are largely favourable due to their tropical climate, wet weather, increased rainfall, wind, temperature, and loose soil. Conditions like heavy rains, floods, storms and cyclones favor the clustering of melioidosis. These uncontrollable natural disasters are frequent in South Asian countries.^26^ Melioidosis is common in areas where agriculture and farming like paddy cultivation are common; construction sites also increases the risk of exposure.^21^ These pathogenic bacteria generally remain in the soil. During the rainy season, the increased soil porosity allows for bacterial movement from lower soil layers to the1 superficial surface for its multiplication.^27^ The average incubation period of melioidosis is nine days (range 1 to 21 days)but can vary from days to years.^28^ The bacteria typically enter through skin abrasions, but other transmission routes like inhalation, ingestion, and rarely even sexual and perinatal transmission have been reported.^29^ The barefoot workers cultivating in the rice fields and the others exposed to soil are at higher risk of developing melioidosis.^30^^,^^31^ Global warming and urbanization increase the incidence of melioidosis and geographical spread.

Although the disease has varied presentation, acute melioidosis typically manifests as pneumonia, sepsis, and multiorgan abscesses. The mortality can be prohibitively high, but it is potentially curable. The actual mortality rates vary across the regions from 9 - 70%.^32^ With an early diagnosis, appropriate antibiotic administration, and advanced intensive care unit, mortality can be decreased to less than 10%.^33^

### Call for measures

#### Increasing awareness for melioidosis

**Figure 3:**
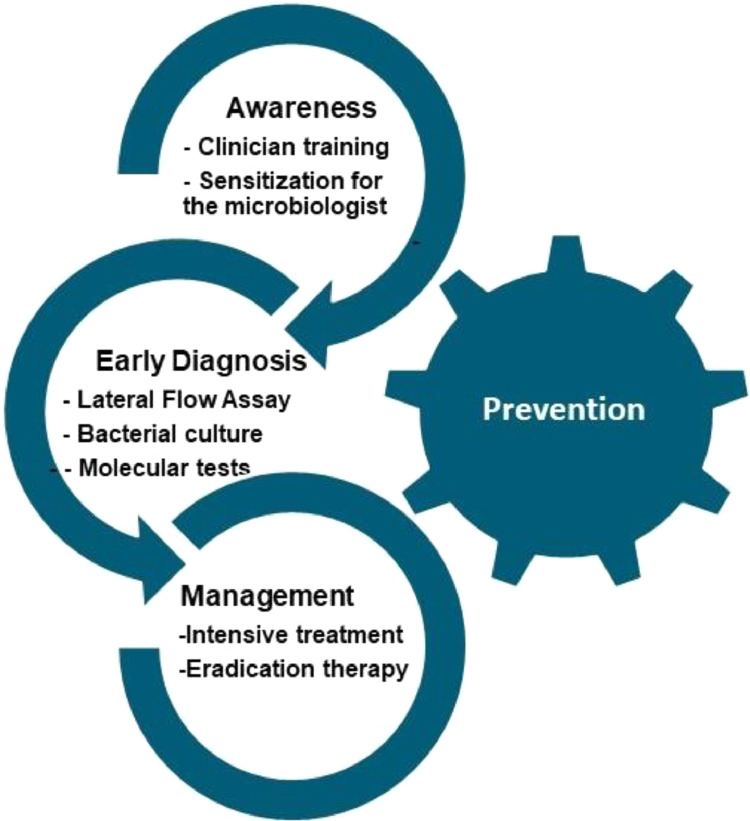
**Key approaches for melioidosis control.** Increasing awareness among clinicians and microbiologists, early point-of-care diagnosis, appropriate case management, and disease prevention are fundamental approaches for controlling melioidosis.

Melioidosis is grossly underreported in India due to its variable non-specific clinical manifestations, limited awareness of the clinical presentation of the disease and misidentification of *B. pseudomallei* with other organisms by inexperienced laboratories. A dedicated group of medical educators should initially increase the awareness by conducting a series of symposiums and workshops for clinicians and microbiologists. Hence, knowledge and awareness among clinicians and clinical microbiologists are imperative for tackling this endemic, poorly recognised, fatal disease. Key approaches for melioidosis control are summarised in Figure 3.

### Early diagnosis

Melioidosis can be treated without difficulty if diagnosed at an early stage. The fatality of melioidosis is due to delay in diagnosis and due to challenges in laboratory identification of *Burkholderia pseudomallei*. Peripheral hospitals may utilize point of care tests of antigen detection. A lateral flow immunoassay (LFIA) that detects *B. pseudomallei* capsular polysaccharide (CPS) via a monoclonal antibody is gaining popularity as a point of care (POC) test^34^ An initial laboratory study using culture-proven bacteria has shown a sensitivity of 98.7% and a specificity of 97.2%.^34^ Overall sensitivity in the diagnosis of melioidosis was 91% from our institutional study.^35^ The LFIA had an average specificity of nearly 100%.^35^ There is a need for further innovation and quality lateral flow assay(LFIA) in all the regions. Some have adopted culture as the only method of diagnosing their cases. LFIA takes a few minutes and is easy to perform as a POC test which detects the capsular polysaccharide antigen of *B. pseudomallei* directly from several tissue samples. This easy method can be adopted while waiting for the culture diagnosis.^35^

In acute bacteremic melioidosis, the disease spreads by hematogenous seeding leading to multiorgan abscesses, which can be assessed by ultrasonography and CT scan. The lung is commonly involved, and chest radiographs show multifocal nodular, patchy consolidation with cavitary lesions.^36^ There may be rapidly progressive patchy consolidation and enlarging coalescent nodules, breakdown, and thick-walled cavitation.^36^ The lung consolidation can be in any lobe of the lung, progressing to abscess and cavity formation that mimics tuberculosis.^36^ The importance of an early diagnosis cannot be overemphasised.

The appropriate treatment regimen includes an induction and maintenance course, which is quite different from other bacterial diseases. The patients require antibacterial treatment with meropenem or ceftazidime injection for the appropriate duration, followed by a long (6 months) eradication therapy.

#### Melioidosis registries

International Melioidosis Network (IMN) is a web-based open networking group started with the initiative of Mahidol University, Thailand.^6^ Healthcare is a national priority of each nation. Sustained political commitment from respective stakeholders and mandatory country-wide reporting, melioidosis disease notifications should document the substantial burden and brings opportunities to alleviate public health risks. Reporting subjects aims to understand case distribution, morbidity, and mortality. Intensified case finding and case management could reduce the disease burden. There is a need for country-specific epidemiological melioidosis databases or registries as a neglected tropical disease. Such epidemiological studies would provide information on a better understanding of disease transmission and preventable risk factors.^37^

### Prevention

Like any other infectious disease, prevention of infection remains a precious strategy. Infection usually starts locally by inoculation, inhalation, or ingestion from environmental sources. The disease is caused after exposure to *B. pseudomallei* in a susceptible person through contact with cracked or damaged unprotected skin, less commonly by aerosol inhalation and contaminated water.^38^ Therefore, basic public health measures include the primary provision of safe drinking water and sanitation, prevention of contact with contaminated soil or water, early case detection and appropriate management, including eradication therapy (to prevent relapse).^15^ Other public health management approaches for increasing awareness and reducing exposure or contamination are information, education, communication (IEC) activities for community education. Health education on the menace and prevention of ailments through social media and school is another measure, particularly in endemic areas. The modes of transmission and important modes of preventions are mentioned in Table-1.

**Table 1:** Mode of transmission and prevention of melioidosis.

It is essential to increase the use of protective, water-resistant 'knee-high boots' in the paddy field and gloves when working outdoors to protect from rain, loose soil, and mud, where the bacteria reside. Precautions should be taken, especially during the rainy season and for farmers working in water-filled wet areas like rice fields.^39^

In Australia, groundwater (bore water) had shown contamination with *B. pseudomallei* in 33% of places. UV irradiation has been shown to decrease the burden of Burkholderia in the domestic water supply to an undetectable level.^40^^,^^41^ Additionally, chlorination of potable water is also an effective disinfection method to control melioidosis.^42^^,^^43^ Large-scale chlorination of water has shown to be effective in Australia despite theoretical concerns about the survival of *B. pseudomallei* in them.^42^ In low-income and middle-income countries, where ultraviolet light treatment of water is not feasible, people must use traditionally boiled water before consumption.^40^ Preventive steps are most important for people with high-risk groups.

The multimodal approach for primary prevention of diabetes and glycaemic treatment of type 2 diabetes would possibly reduce the overall problem of melioidosis more effectively than any of the above five strategies to prevent neglected tropical diseases.

## Conclusions

Though described 26 years ago as the tip of the iceberg, Melioidosis continues to expand in India. The disease burden is likely to increase further due to the increasing prevalence of diabetes and population growth in South Asia. With the vast population and rising burden of melioidosis in South Asia, insufficient diagnostic laboratory facilities remain the key challenge to address morbidity and mortality. Establishing an effective prevention program is a formidable challenge in the vast South Asian situation. Improved awareness and understanding of transmission dynamics should facilitate enhanced surveillance, early diagnosis, case management and preventive interventions. The burden of melioidosis reveals the gaps in dealing with the disease, whereas the prevention mechanism clearly demands public health action in South Asia. Without early diagnosis and treatment and exploring better ways to prevent transmission, melioidosis will spread exponentially and claim more lives in the years to come.

## Contributors

PM conceptualised the idea for the article, performed the literature search, wrote the initial manuscript. BM expanded ideas for the article, modified the search, wrote the final form of the manuscript.

## Declaration of interests

The authors declare no conflict of interests.
